# Biogenic production of silver, zinc oxide, and cuprous oxide nanoparticles, and their impregnation into textiles with antiviral activity against SARS-CoV-2

**DOI:** 10.1038/s41598-023-36910-x

**Published:** 2023-06-16

**Authors:** David Asmat-Campos, Jesús Rojas-Jaimes, Gabriela Montes de Oca-Vásquez, R. Nazario-Naveda, D. Delfín-Narciso, L. Juárez-Cortijo, Damaris Esquen Bayona, Benoit Diringer, Reinaldo Pereira, Diego Batista Menezes

**Affiliations:** 1 Dirección de Investigación, Innovación y Responsabilidad Social, Universidad Privada del Norte (UPN), Trujillo, Peru; 2 National Laboratory of Nanotechnology, National Center for High Technology, Pavas, San José 10109 Costa Rica; 3 Grupo de Investigación en Ciencias Aplicadas y Nuevas Tecnologías, Universidad Privada del Norte (UPN), Trujillo, 13011 Peru; 4 INCABIOTEC SAC, Tumbes, 24 000 Peru; 5 Ingenetic Solutions S.A.C, Lima, Peru; 6 Programa de Maestría de Biotecnología Molecular, Universidad Nacional de Tumbes, Tumbes, 24 000 Peru

**Keywords:** Cell biology, Genetics, Molecular biology, Materials science, Nanoscience and technology

## Abstract

Nanotechnology is being used to fight off infections caused by viruses, and one of the most outstanding nanotechnological uses is the design of protective barriers made of textiles functionalized with antimicrobial agents, with the challenge of combating the SARS-CoV-2 virus, the causal agent of COVID-19. This research is framed within two fundamental aspects: the first one is linked to the proposal of new methods of biogenic synthesis of silver, cuprous oxide, and zinc oxide nanoparticles using organic extracts as reducing agents. The second one is the application of nanomaterials in the impregnation (functionalization) of textiles based on methods called "in situ" (within the synthesis), and "post-synthesis" (after the synthesis), with subsequent evaluation of their effectiveness in reducing the viral load of SARS-CoV-2. The results show that stable, monodisperse nanoparticles with defined geometry can be obtained. Likewise, the "in situ" impregnation method emerges as the best way to adhere nanoparticles. The results of viral load reduction show that 'in situ' textiles with Cu_2_O NP achieved a 99.79% load reduction of the SARS-CoV-2 virus.

## Introduction

Humanity is facing one of the worst health catastrophes reported in recent decades, coronavirus disease 2019 (COVID-19)^1^. COVID-19 is caused by the type 2 coronavirus causing severe acute respiratory syndrome (SARS-CoV-2), which has spread worldwide through multiple modes of transmission, such as direct contact or airborne particles^2^. Consequently, government agencies worldwide have considered various health measures aimed at curbing contagion, adopting measures ranging from social distancing to the use of safety and protective equipment such as masks and face shields^3–5^. However, although face masks have become essential for interactions in public spaces, their prolonged use without disinfection, contact with contaminated surfaces, and improper disposal in landfills that pollute the environment make them a global challenge^6–10^.

Reusable masks significantly reduce the pollution generated by disposable synthetic masks^11^. However, they must go through a constant disinfection and washing process, which makes them impractical for healthcare environments. Studies indicate that SARS-CoV-2 remains active on organic and non-organic surfaces, which emphasizes a complex health context driving the development of biocidal materials^12–14^. Thus, for example, wound dressings based on the impregnation of silver nanoparticles (Ag NP) in wool, cotton, viscose, nylon, and polyamide were analyzed, confirming the antibacterial properties of silver as an effective functionalization system for these textiles. This property was reconfirmed in the assessment of a bandage by impregnation of cotton fabric with crystalline Ag NPs and bimetallic Ag/Cu compounds attributing antibacterial and antifungal properties to bimetallic fabrics which were tested on a wide range of multidrug-resistant bacteria and fungi such as *Enterobacter aerogenes, Proteus mirabilis, Klebsiella pneumoniae, Candida albicans* and micromycetes^15–18^.

In that sense, nanotechnology is being used to fight off infections caused by viruses, and the design of protective barriers such as face masks is among the most prominent nanotechnological uses^19^. Copper (Cu NP), Ag NP, and gold (Au NP) nanoparticles could be involved in the production of proinflammatory cytokines and Th1 response with antiviral activity^20^. Nanomaterials (NMs) are relevant in the fight against the SARS-CoV-2 virus, the causative agent of COVID-19^21^. These NMs can inhibit viral entry by binding to the viral receptor known as the angiotensin conversion enzyme (ECA-2), preventing the escape of the endosome^22^. The use of NP or antiviral substances in a fabric matrix is important for its application on clothing or masks with antiviral effects, as demonstrated in a previous study^23^.

Ag NP has demonstrated an antiviral effect (Influenza A, Hepatitis B, and HIV-1) penetrating virus membranes and interacting with the viral genome, preventing replication. This antimicrobial effect has been related to the NP size^24^. In addition, Ag NP has demonstrated an antiviral effect against feline coronavirus, MERS-CoV, and SARS-CoV-2^25–27^. In the case of SARS-CoV-2, up to 21.5% inhibition was demonstrated with a low level of toxicity^27^.

In addition, it has been determined that Ag NPs can inhibit HIV. These nanoparticles interact with the virus surface glycoproteins, interfering with the viral binding and entry into host cells^28^. Likewise, another study shows that Ag NPs are highly toxic to the gamma-herpes virus, known as Kaposi's sarcoma-associated herpesvirus (KSHV). These NPs block primary KSHV infection destroying virion particles directly, inhibiting colony formation effectively, and suppressing the growth of KSHV-associated tumors moderately^29^.

Apart from Ag NPs, protein nanofibrils and iron oxyhydroxide nanoparticles were used to create a filtration membrane which shows an efficiency of more than six orders of magnitude reduction in the infectivity of H1N1 and SARS-CoV-2 viruses, demonstrating that this membrane composed of nanomaterials not only retains the virus but can also inactivate them^30^. Studies have also shown that Copper (Cu) ions as well as Zinc (Zn) interact with different viral components such as proteins, which can be inactivated when replaced with Cu. This is because proteins tend to select divalent metal ions from the environment, following the Irving-Williams order of stability (Mg ^2+^  < Mn ^2+^  < Fe ^2+^  < Co ^2+^  < Ni ^2+^  < Cu ^2+^  > Zn ^2+^)^31,32^, finding that Cu damages the viral genome of HIV and Herpes Simplex Virus (HSV) by inhibiting proteases when it interacts with the envelope cysteine.

The principle of SARS-CoV-2 transmission is based on using angiotensin-converting enzyme 2 (ACE2) and proteases as entry activators by fusing the viral membrane with the host cell membrane to eventually achieve invasion, so proposals to inactivate this interaction would lead to an inhibition of virus transmission^33^. Coronavirus inactivation on copper and copper alloy surfaces resulted in a nonspecific and irreversible fragmentation of RNA, with fragments becoming smaller as contact time increases^34^.

Proposals to deal with and reduce the spread of the coronavirus include the use of nanotechnology, and, among these options, the functionalization of textiles with NPs promises to be very useful against dangerous microorganisms. An evaluation of SARS-CoV survival between two hospital safety gowns made of different textiles, a disposable fluid-repellent one and a cotton one, found that the cotton material immediately absorbed highly concentrated droplets of virus culture and, one hour later, no active virus was detected, despite the organic base of the gown material. However, most of the virus remained active in disposable gowns for up to 24 h^35^. This is in relative contrast to the study conducted under conditions of 21–23 °C temperature and 40% relative humidity in which a higher persistence of both SARS-CoV-1 and SARS-CoV-2 coronavirus on cardboard (organic material) was evaluated compared to non-absorbent materials such as plastic and stainless steel^36^. Very recently, reusable face mask designs have also made use of new technologies to improve their functionality regarding their antimicrobial, fungicidal, or viricidal properties, seeking to neutralize SARS-CoV-2 by reducing its permanence on surfaces such as gowns, uniforms, and any fabric used for health care, enhancing the functionality of protective equipment. However, the new proposals are still being evaluated to meet the requirements of safety, economy, efficacy, and sustainability^37–39^.

This study presents novel methods for the biogenic synthesis of silver nanoparticles (Ag NPs), zinc oxide nanoparticles (ZnO NPs), and cuprous oxide nanoparticles (Cu_2_O NPs), which are subsequently applied in the functionalization of textiles. It is also important to consider the evaluation of the type of mechanism used in textile impregnation, a topic that is extensively discussed in this research. Finally, the antiviral activity of the textiles against the SARS-CoV-2 virus was evaluated.

## Methodology

The reagents used for the synthesis of metal nanoparticles were purchased from Merck Millipore, and ultrapure water (Thermo Scientific, Barnstead Smart2Pure, MA, USA) was used throughout the experiment. Likewise, the textile used in all the experiments contains 70% cotton and 30% polyester, with a weft-type configuration and taffeta construction. The functionalized textiles were stored in properly sterilized Kraft paper packaging for later use.

**Figure 1:**
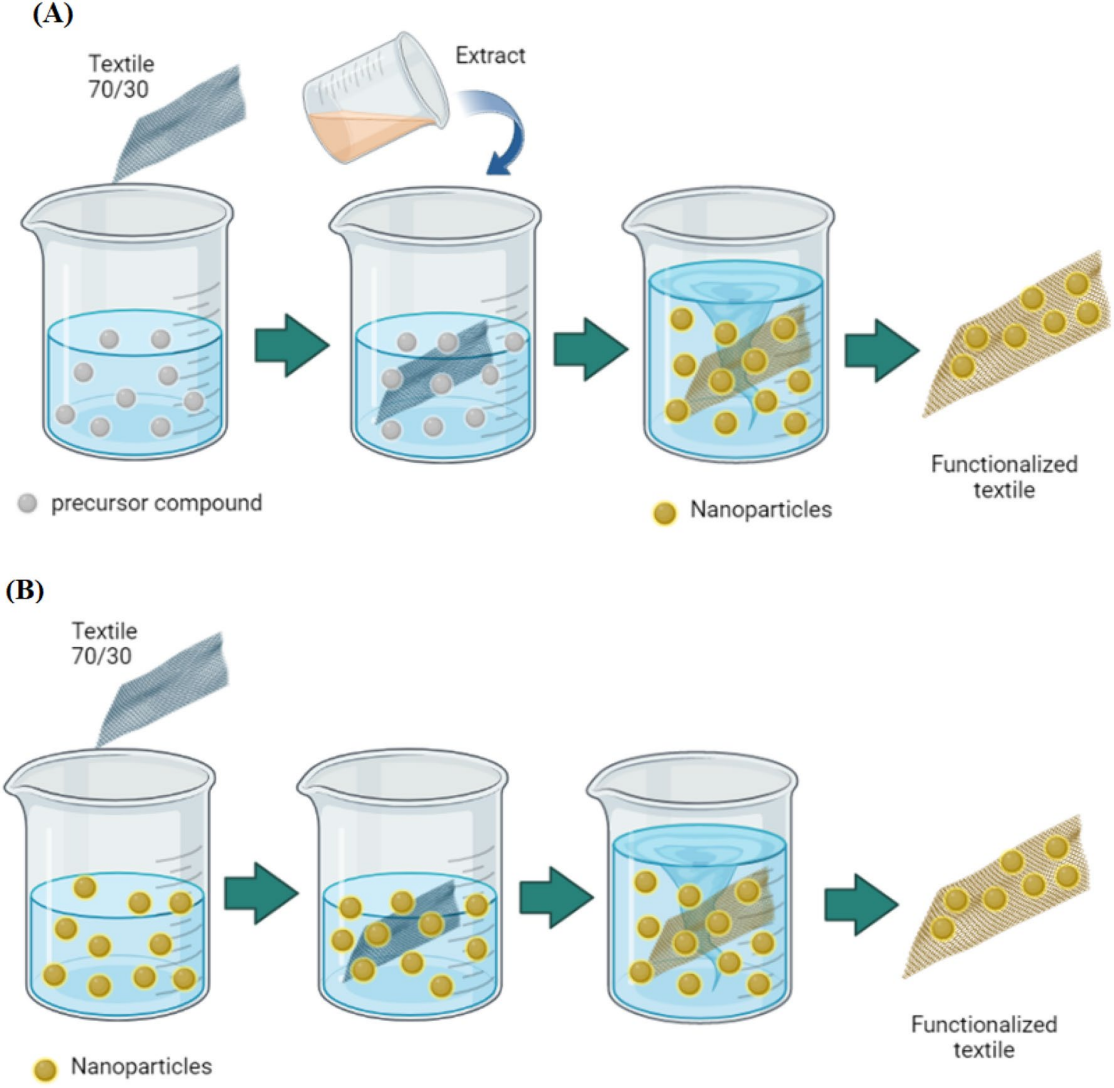
Method of impregnation of nanomaterial in textile: (**A**) "In Situ" and (**B**) "Post-synthesis".

Two methods of nanoparticle impregnation into the textile were evaluated: "in situ" (Fig. 1A) and "post-synthesis" (Fig. 1B).

### Biogenic synthesis of silver nanoparticles (Ag NP) and their impregnation in textiles by "in situ" and "post-synthesis" methods

For the synthesis of AgNP and their impregnation in textiles by the "in situ" method, a stock solution [1M] of the ACS silver nitrate precursor (CAS No. 7761-88-8) was used. On this basis, 500 µL of the precursor was extracted and diluted with ultrapure water to 500 mL, which was kept under magnetic stirring (400 rpm) until reaching 60 °C. Once the temperature was reached, the textile piece was immersed and kept for 5 min. Then, a burette was filled with 25 mL of 96% alcoholic extract of *Eucalyptus globulus* (previously filtered); then, it was added drop by drop to the precursor solution maintaining the same temperature and agitation for 5 min. Next, sodium hydroxide ACS (NaOH) (CAS no. 1310-73-2) was added until the solution reached a pH of 10, maintaining stirring for 20 min. Once the procedure was completed, the textile sample was removed and left to drain for 10 min. Finally, the sample was placed in a forced convection oven (100 °C) for 15 min.

For the 'post-synthesis' impregnation of Ag NP in textile, the same biogenic synthesis protocol described above was developed (without immersing the textile sample in the middle of the process). In this sense, 500 mL of Ag NP colloid was heated up to 60 °C and, then, the textile piece was immersed and kept for 20 min with magnetic stirring (400 rpm). Next, the sample was removed, washed with acetone and distilled water, and left to drain for 10 min. Finally, it was placed in a forced convection oven (100 °C) for 15 min.

### Biogenic synthesis of cuprous oxide nanoparticles (Cu_2_O NP) and impregnation in textiles by in situ and post-synthesis methods

The Cu_2_O NP synthesis was mediated by the green route. For this purpose, the juice of *Myrciaria dubia* "camu camu" was used as a reducing agent. In this case, the copper sulfate pentahydrate precursor (CuS0_4_5H_2_O) was used, preparing 450 mL at 0.05 M. The mixture was kept under magnetic stirring (400 rpm) for 10 min at room temperature. Next, the textile piece (70% cotton/30% polyester) was immersed and kept under stirring for 3 min. Then, 22.5 mL of 7.5 M sodium hydroxide (NaOH) was added. At this stage, the stirring was increased to 1200 rpm. The reduction process occurred when 90 mL of *M. dubia* juice was added dropwise under the same temperature and agitation conditions. The sample was kept for 10 min. Finally, it was removed, drained for 10 min, and placed in a forced convection oven (100 °C) for 15 min. This method corresponds to "in situ" textile impregnation.

For "in situ" textile functionalization, Cu_2_O NP was synthesized using the same protocol described above but without considering adding the textile during the process. In this case, 500 mL of colloid was used, in which the textile piece was immersed and kept for 60 min with magnetic stirring (1200 rpm) at room temperature. Finally, the sample was removed, washed with acetone and distilled water, and allowed to drain for 10 min before being placed in a forced convection oven at 100 °C for 15 min.

### Biogenic synthesis of zinc oxide nanoparticles (ZnO NP) and impregnation in textiles by "post-synthesis" method

The starting point for the synthesis of ZnO NP was the zinc acetate ACS precursor (CAS No. 5970-45-6). For this, 30 mL of the precursor at 0.21 M was prepared. The reaction was carried out on a hotplate until a temperature of 70 °C was reached using magnetic stirring at 450 rpm. Next, 20 mL of aqueous extract of *Coriandrum sativum* was added with magnetic stirring (600 rpm) for 4 h. Finally, the sample was calcined in a muffle for 2 h at 500 °C.

The textile treatment was only for the "post-synthesis" process because the ZnO NP must go through a calcination process, which makes it impossible to do the treatment during the formation of nanostructures. In this case, 0.21 g of ZnO NP powder was diluted in 350 mL of ultrapure water and homogenized in ultrasound for 30 min at room temperature. Subsequently, the colloid was poured into a beaker and heated to 70 °C. The textile sample was then completely immersed and kept under magnetic stirring (400 rpm) for 1 h. Next, the sample was washed with acetone and distilled water, and left to drain for 10 min. Finally, the textile was placed in an oven at 100 °C for 15 min.

### Textile treatment with Ag/Cu_2_O nanoparticles by "post-synthesis" method

The silver nanoparticles (Ag NP) and cuprous oxide nanoparticles (Cu_2_O NP) were synthesized separately based on the previously described method. The nanoparticles were impregnated by the "post-synthesis" method due to the nature of the final state of the colloids. For this purpose, the textile sample was immersed in 500 mL of Ag NP and kept in constant stirring (600 rpm) for 60 min at room temperature. Then, it was removed and left to drain for 10 min. Finally, the sample was dried in a forced convection oven (100 °C) for 15 min. The sample obtained, and previously impregnated with Ag NP, went through the impregnation with Cu_2_O NP, immersing the textile sample in 500 mL of Cu_2_O NP colloid for 60 min under constant stirring (1200 rpm), to finally remove it, let it drip-dry for 10 min and wash it with acetone and distilled water. Finally, the textile sample was dried in a forced convection oven at 100 °C for 15 min.

### Characterization of nanoparticles

The NPs were initially characterized by UV–vis spectrophotometry (UV 1900, Shimadzu) in the range of 200–900 nm. The size and shape of the NPs were analyzed through transmission electron microscopy (TEM) by adding 5 µl of the colloid placed on a carbon-coated copper grid. Then, the NP sample was dried in a desiccator with silica for 16 h. Measurements were carried out on a JEOL (model JEM 2011) powered with a voltage acceleration of 120 kV. EDX mediations were carried out with the TEM equipped with an OXFORD EDS 6498. The crystal structure was analyzed by X-ray diffraction with a Cu Kα source in the 2θ range of 20–80°. FTIR analysis was performed to determine the main functional groups present in the NPs due to the presence of traces of the extracts. FTIR spectrum was collected at 4 cm^−1^ resolution and in the transmission mode (4000–500 cm^−1^) using an FTIR spectrophotometer (Nicolet 6700, Thermo Scientific). The spectra were analyzed with OMNIC 8.1 software (OMNIC Series 8.1.10, Thermo Fischer Scientific).

### Obtainment of synthetic RNA from the E gene of the SARS-CoV-2 virus

Twenty positive samples with a Cq between 15 and 20 were selected. Subsequently, RNA was extracted using the GeneAid viral RNA Mini kit. The RNA obtained was quantified with digital equipment (NanoPhotometer ® NP80) and stored at − 80 °C.

RNA was treated with DNAase I, Thermo Fisher (#EN0521), and retrotranscribed using 10 µM oligodT, following the recommendation of Thermo Scientific RevertAid RT Kit (#K1691). Subsequently, PCR was performed to obtain the amplicons with the T7 polymerase binding site, using primers TIVF3 (TAATACGACTCACTATAGGGACCGACGACGACGACGACTACTAGC) and B3 (AGAGTAAACGTAAAAAGAAGAAGGTT) which were migrated on 1.8% agarose gel. The amplicons were purified with Beckman Coulter AMPure XP microbeads—REF A63880, following the manufacturer's recommendations. RNA synthesis of the E-gene target region was performed using the HiScribe™ T7 Quick High Yield RNA Synthesis Kit for in vitro transcription from the purified amplicons. The in-vitro transcribed RNA was treated with DNAase I and purified using the Monarch® RNA Cleanup Kit T2040L, according to the manufacturer's recommendations. Finally, the RNA was quantified with a Qubit fluorometer.

### Inoculum preparation

Twenty positive samples with Cq between 14–30 were selected from oro-nasopharyngeal swabs preserved in UTM (universal transport medium). 500 µL of each sample were mixed to obtain a 20 mL pool; a 30-s vortexing and spin-down were performed; 100 µL of this pool was aliquoted to evaluate the Cq; and the number of copies was identified. RNA was extracted using the GenElute™ Universal Total RNA Purification Kit, following the manufacturer's recommendation.

### RT-qPCR standard curve for inoculum assessment

The standard curve was performed from RNA synthesized in vitro, using serial dilutions (1/10) with three replicates for each dilution. The number of copies was determined considering the molecular weight of the ssRNA sequence of the target region. In addition, the inoculum was assessed, considering 5 µL of extracted RNA. For RT-qPCR, the CAPITAL™ qRT-PCR Probe Mix kit was used, 4 × , 0.4 µM of primers E-Sarbeco-F1 and E-Sarbeco-R2, 0.2 µM of E-Sarbeco-P1 Probe, 0. 8 X of qPCR Probe Mix and 1 µL of Rtase with RNAse inhibitor, applying 50 °C reverse transcription for 10 min, followed by 95 °C denaturation for 3 min and 45 cycles of 95 °C for 10 s and 58 °C for 30 s^40^. The amplification process was performed on the mic qPCR thermal cycler, using probe hydrolysis as the chemistry type, selecting the FAM fluorochrome and the BHQ-1 Quencher, and activating the green channel in the elongation step.

To read the data, the threshold value was set, and the NTC (non-template control), CE (extraction control), and CP (positive control) controls were evaluated as comparison standards for the study samples. Finally, the equipment was configured to obtain the standard curve, and the Cq value of the inoculum was read and extrapolated to determine the number of copies.

### Inoculation of functionalized textiles with SARS-CoV-2

150 µL of SARS-CoV-2 inoculum was applied to two fabrics (70% cotton and 30% polyester) with the In Situ functionalization process with Ag NP (Ag-2,411 ppm) and Cu_2_O NP (Cu_2_O-734, 132 ppm), and four fabrics with *Post Synthesis* process with Ag NP (Ag-2,411 ppm), Cu_2_O NP (Cu_2_O-734,132 ppm), silver/copper (Ag/Cu_2_O 2.41 Ag ppm/734.13 Cu ppm) and ZnO NP (ZnO 398.7 ppm). Only the fabric with 70% cotton and 30% polyester without NP was used as a control. Three replicates of each NP-treated fabric were made.

The inoculated fabric was incubated at 18 °C for 20 h. Then, the inoculated area was swabbed; the swab end was placed in 1.5 mL tubes, and RNA was extracted with the GenElute™ Universal Total RNA Purification Kit, following the manufacturer's recommendation. RT-qPCR was performed as explained above. The viral readout was performed considering nanogram/µL concentration, which was then translated to the number of copies. The percentage of viral reduction was determined according to the equation:

$$\%{R}_{v}=\left[\frac{B-A}{B}\right]100$$

where B = the viral load of the inoculum, and A = the viral load of the NP treatment of the fabric.

### Statistical analysis

The data represent the mean of three independent experiments and the standard deviation. Statistical comparisons were performed by one-way analysis of variance (ANOVA). All p-values less than 0.05 were considered statistically significant.

## Results

### Biogenic synthesis of silver nanoparticles (Ag NP)

**Figure 2:**
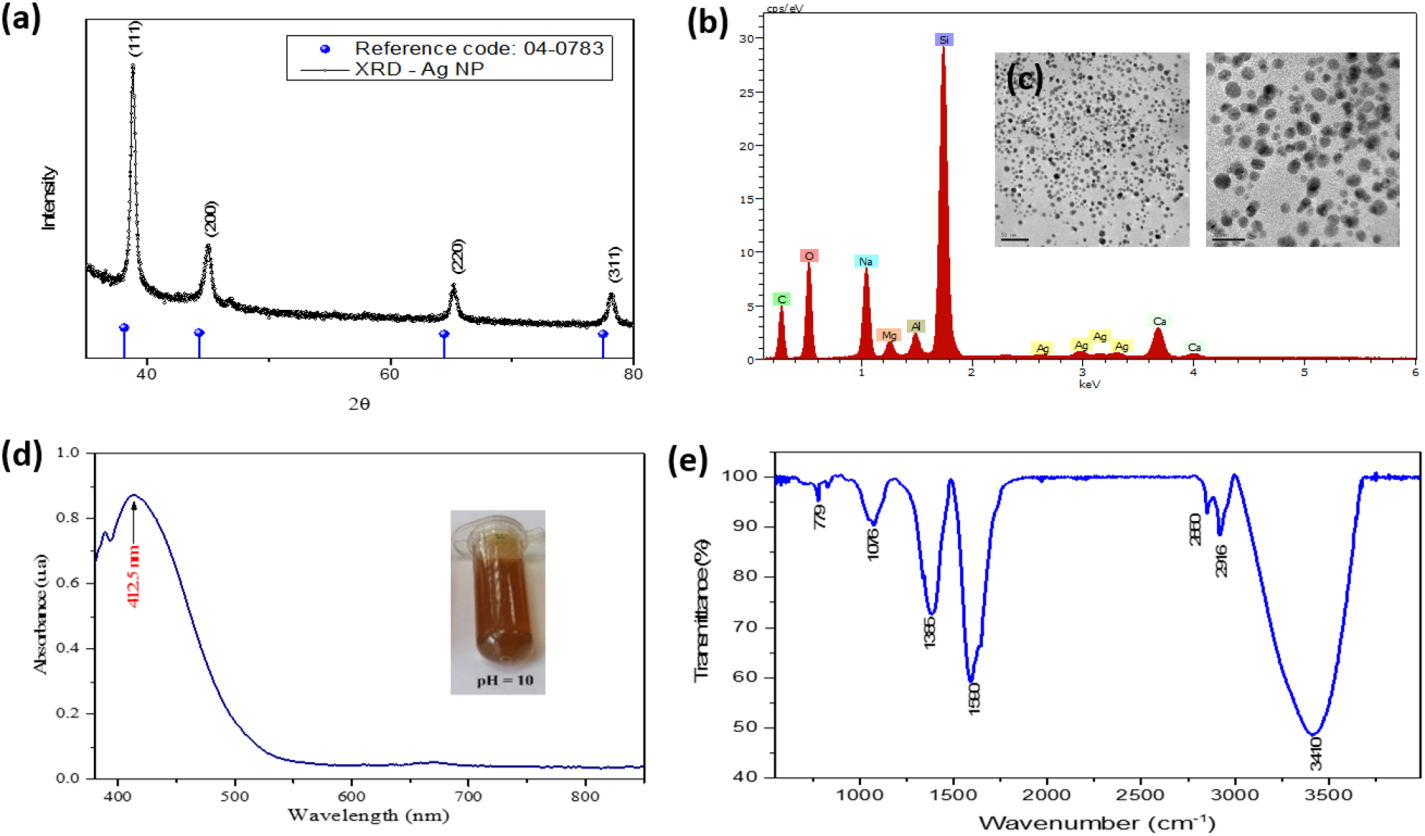
Characterization of Ag NP. (**a**) X-ray diffraction, (**b**) EDS elemental analysis, (**c**) TEM, (**d**) UV–vis spectrophotometry, (**e**) FTIR.

Figure 2a shows the X-ray diffraction (XRD) patterns for the colloidal Ag NP. Two peaks can be observed at the 38.1°and 43.5° positions corresponding to the (111) and (200) planes of silver as indicated by the JCPDS card, No. 04-0783. The results confirm that the Ag NPs are cubic crystals centered on the faces by the intensity of the (111) peak^41,42^.

The morphology and size of the synthesized Ag NP were analyzed by TEM. The images shown in Fig. 2b indicate that the NPs have mostly spherical shapes, and only a minority have various polygonal and elongated shapes with an average size of 6 ± 3.7 nm. The NPs appear scattered with small agglomerations of various sizes^43,44^. EDS analysis shown in Fig. 2c confirms the formation of Ag NP.

Another technique used to confirm the formation and stability of the synthesized AgNPs was UV–vis spectrophotometry. Figure 2d shows the UV–vis absorption spectrum where a characteristic band for this material is observed, with its peak located at 412.5 nm. One of the authors of this research^45^ developed a synthesis method for this type of nanomaterial via chemical route, obtaining a plasmon peak at 434 nm. Applying the Mie theory, the nanoparticle size was determined to be 59.2 nm. In contrast, this research shows a plasmon resonance peak at 412.5 nm, indicating a shift towards shorter wavelengths, suggesting a decrease in the effective size of the silver nanoparticles. This is corroborated by the average size result mentioned above. The results suggest a good reduction process and formation of nanostructure obtained by the biogenic route method.

FTIR spectra were used to identify the biomolecules possibly responsible for reducing silver ions for NPs formation in the synthesis process. The FTIR spectra show various functional groups that are related to organic molecules. Figure 2e shows this spectrum for a sample of Ag NP in a colloidal state and bio-reduced with eucalyptus extract. The broad and long peak located at 3411 cm^−1^ and the peak at 2916 cm^−1^ correspond to the hydroxyl (–OH) bonds by the vibration of the alcohol components and to the aldehyde C-H group by the vibration of the alkane components. The peak at 1591 cm^-1^ could correspond to the vibrations of the C–N bonds of the carboxyl group (–C=O). The peaks located at 1386 cm^−1^ and 1077 cm^−1^ correspond to the interactions of N–H amide and –C–N amine bonds because of possible silver nitrate waste. The lowest peak located at 780 cm^−1^ corresponds to small vibrations of alcohol bonds (O–H) associated with carboxyls and hydroxyls, which could participate in the synthesis process of NPs^46^.

### Biogenic synthesis of cuprous oxide nanoparticles (Cu_2_O NP)

**Figure 3:**
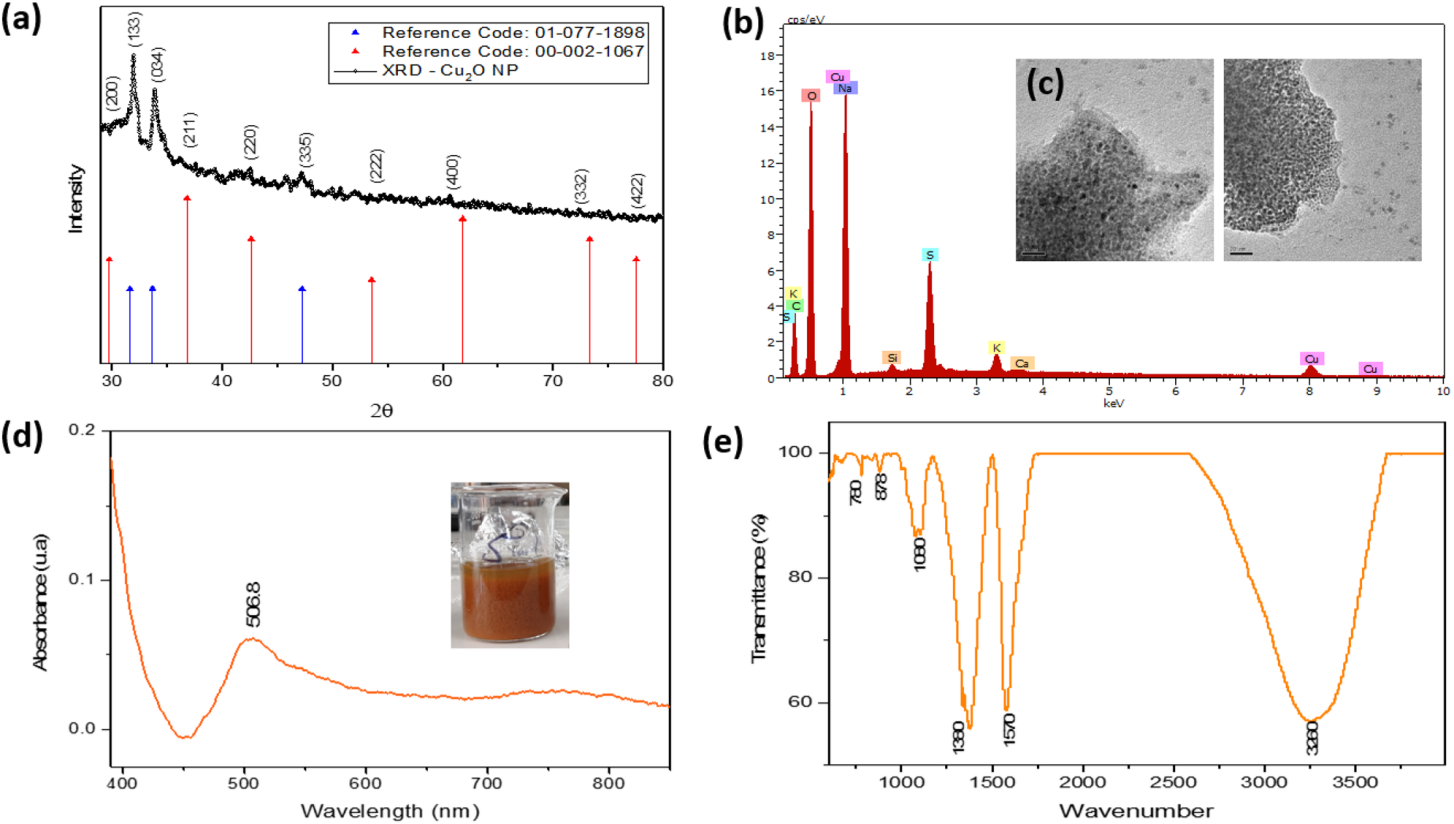
Characterization of Cu_2_O NP. (**a**) X-ray diffraction, (**b**) EDS elemental analysis, (**c**) TEM, (**d**) UV–vis spectrophotometry, (**e**) FTIR.

Figure 3 shows a broad spectrum of characterizations of Cu_2_O NP. X-ray diffraction patterns are shown in Fig. 3a, and the results show high crystallinity with noticeable diffraction angles of 23, 25, 28, 32, 34, 43, and 47° corresponding for Cu to a face-centered cubic structure^47,48^.

TEM analysis (Fig. 3c) gives us information on the shape, size, and distribution of the NPs. It was determined that the NPs have irregular shapes and, in some cases, near-spherical shapes, with the formation of agglomerations. The average size of the synthesized Cu_2_O NP is 10 ± 2.92 nm, which is small compared to other publications^49^ and similar to others synthesized by the green route too^50^. It is suggested that the size achieved is due to the influence of the plugging effect of the extract.

EDS analysis provides a quantitative analysis of the elements present in the formation of Cu_2_O NP. Figure 3b shows the presence of Cu but also a significant presence of oxygen, which suggests the formation of oxides in the synthesis process. The other elements are associated with traces derived from the initial reagents and the *M. dubia* extract.

The formation of Cu_2_O NP was also confirmed by UV–vis spectrophotometry analysis (Fig. 3d). The absorption intensity was reached at 506.8 nm, characteristic of Cu_2_O NPs^48,51^. The broadening of the peak indicates polydisperse NPs.

The FTIR analysis is shown in Fig. 3e. The peak found at 3250 cm^−1^ belongs to the –OH groups due to the adsorption of water molecules, although it can also be related to the carboxyl group. At 1580 cm^−1^ there is a peak associated with the –C–N amine group, while the peak at 1380 cm^−1^ represents the presence of alkanes, and the peak at 1080 cm^−1^ indicates the –C–O group. The peak at 880 cm^−1^ represents the absorption of Cu–O–H bonds, indicating that functional groups play an important role in the synthesis of Cu_2_O NP, specifically in the reduction process. These results are similar to those reported by previous works^50,52^ .

### Biogenic synthesis of zinc oxide nanoparticles (ZnO NP)

**Figure 4:**
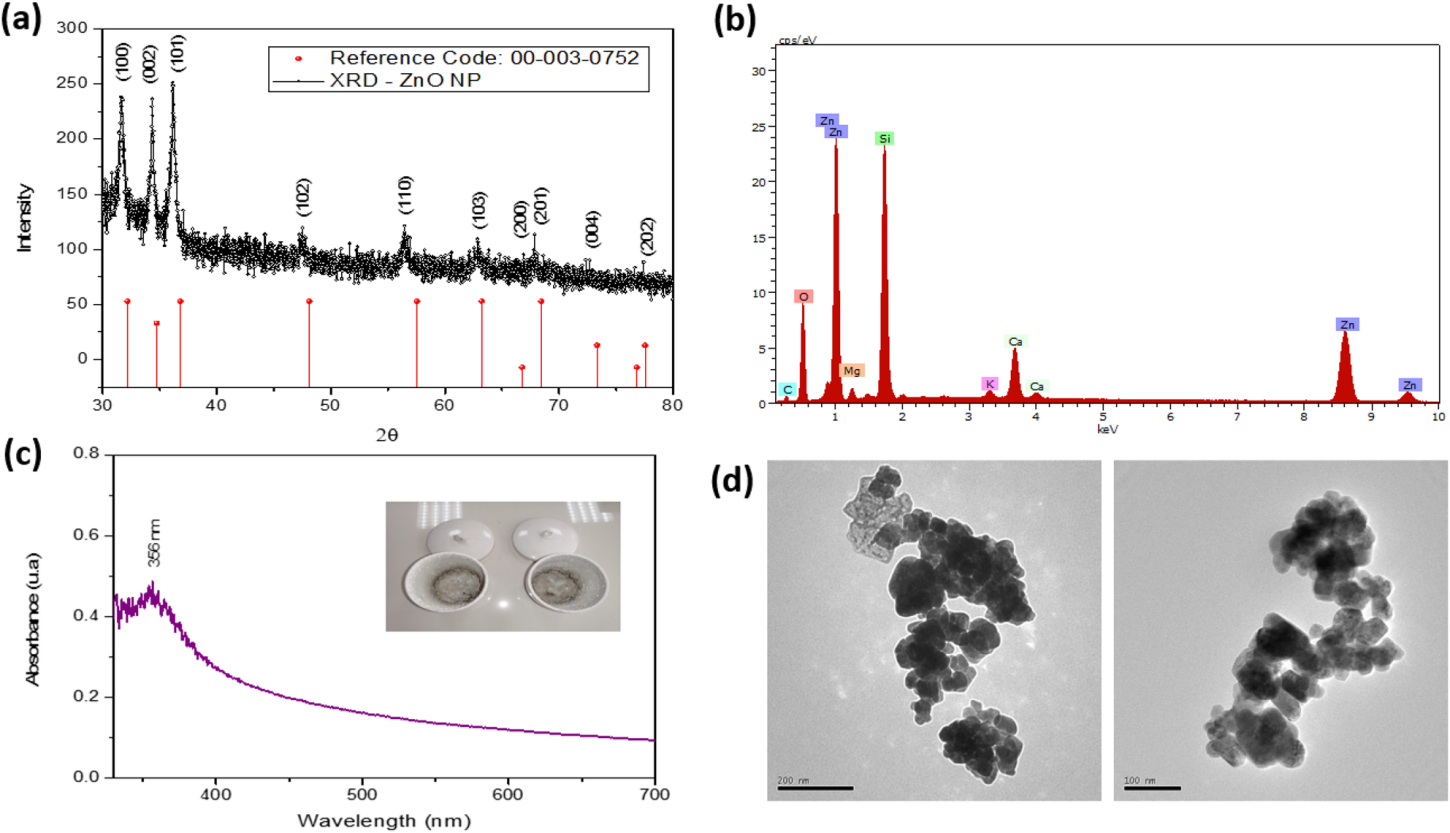
Characterization of ZnO NP. (**a**) X-ray diffraction, (**b**) EDS elemental analysis, (**c**) UV–vis spectrophotometry, (**d**) TEM.

Figure 4 shows the characterization results of the biogenic synthesis of ZnO NP. Figure 4a evidences the diffraction patterns of ZnO NP. All the diffraction peaks correspond to the characteristic hexagonal wurtzite structure and agree with the JCPDS card, No 36-1451^53,54^. The characteristic peaks correspond to the lattice planes (100), (002), (101), (102), (110), (103), and (112), with no peaks due to impurities observed.

Figure 4b shows the EDS analysis confirming the formation of ZnO NP. The UV–vis absorption spectra of the ZnO NPs are shown in Fig. 4c, where the typical absorption peak that corresponds to this material is observed at 355.5 nm. However, a slight blue shift is observed in comparison to that found by other authors^55–57^. Figure 4d shows irregular hexagonal and near-spherical shapes with an average size of 50 ± 8.3 nm clustered in scattered agglomerations, similar to those found by Al-Kordy et al.^58^.

### Textiles functionalized with Ag, Cu_2_O, ZnO and Ag/Cu_2_O nanomaterials

**Figure 5:**
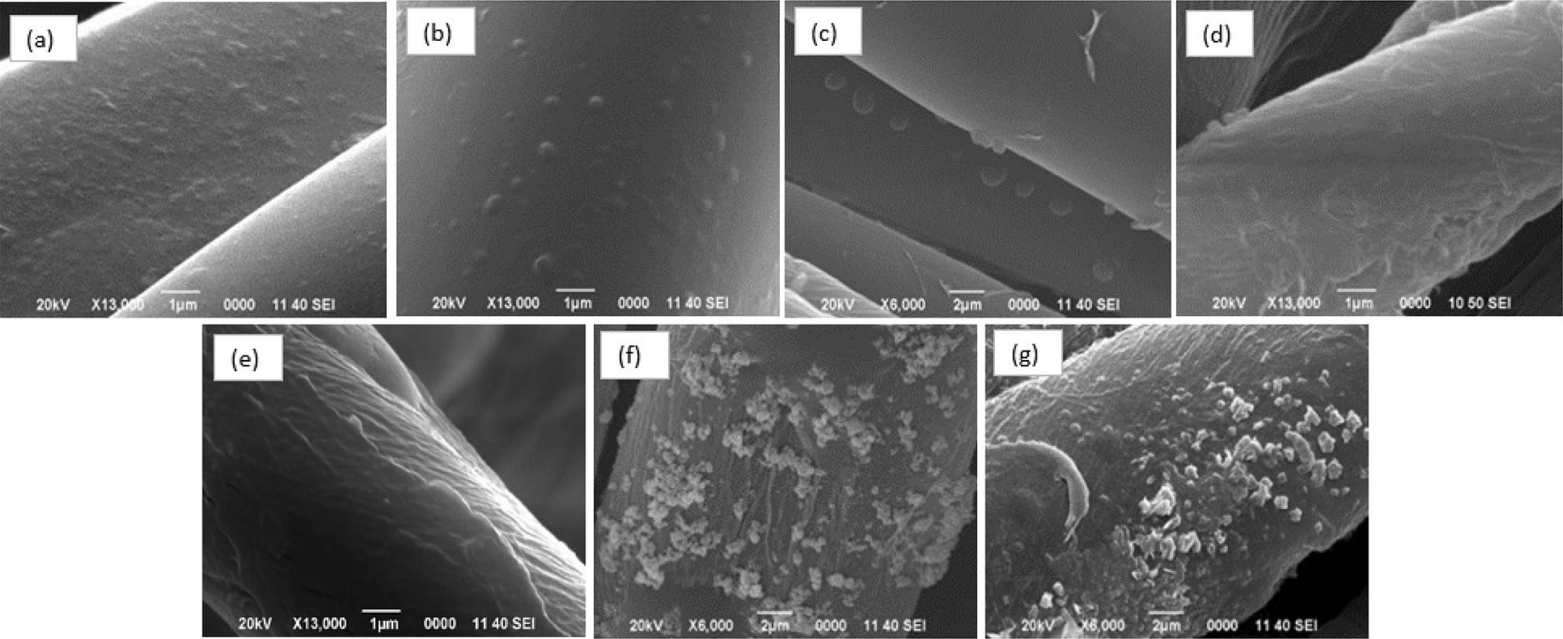
Scanning electron micrograph of the textile fiber functionalized with NP. (**a**) Control, (**b**) In Situ Ag NP, (**c**) Post synthesis Ag NP, (**d**) In Situ Cu_2_O NP, (**e**) Post-synthesis Cu_2_O, (**f**) Post-synthesis ZnO NP, (**g**) Post synthesis Ag/Cu_2_O.

The NPs were impregnated into the textile according to the previously described method. Figure 5 shows the SEM images of the textile fiber, which can be differentiated from the control (Fig. 5a) (without incorporation of NPs). In the case of Ag NP incorporation, as mentioned in the colloid section, quite small nanostructures (6 nm) have been achieved. The "in situ" impregnation method has generated that the NPs are formed in higher percentages in the fiber core and on the surface. Figure 5b represents the Ag NP treatment within situ impregnation, where small agglomerates can be observed. On the contrary, Fig. 5 c shows accumulations of higher quantities because the textile was immersed after having synthesized the Ag NP, finding agglomerations that finally cannot fully penetrate the fiber core, remaining in greater concentration on the surface. In the case of Cu_2_O NP (Fig. 5d), the images show that "in situ" impregnation has over-accumulated the textile fiber with nanomaterial through the presence of multiple layers, especially considering that the NPs had a concentration of 734.13 ppm, i.e., there is accumulation both in the core and on the surface. In the case of "post-synthesis" impregnation (Fig. 5e), it can be noted that there is a non-saturated coating.

The ZnO NPs were obtained as a white powder, which was subsequently dissolved in ultrapure water for the textile immersion process. However, it was possible to observe in Fig. 5f the presence of small agglomerations around the fiber and the textile functionalized with Ag/Cu_2_O hybrid NPs (Fig. 5g), which, being the same method, shows accumulations in large proportions around the fiber.

The application of nanoparticles in the production of textiles has proven to be an effective strategy to improve their properties. However, one of the main challenges in incorporating nanoparticles into textiles is their stability within the textile matrix. In this regard, the stability of nanoparticles in impregnated textiles has been evaluated through various characterization techniques, such as SEM, EDS, and FTIR.

At a morphological level, colloid nanoparticles incorporated into the textile matrix have not undergone any changes. Moreover, energy-dispersive X-ray spectroscopy (EDS) (Figure S1) shows the presence of elements, consolidating proper adherence and no change or reaction due to the presence of polyester material in the textile, ensuring elemental stability.

On the other hand, Fourier transform infrared spectroscopy (FTIR) (Figure S1) is a technique that allows the identification of chemical interactions between nanoparticles and the textile matrix. This technique is based on measuring the absorption of infrared radiation by the sample and is used to determine the chemical characteristics of samples. The results of this technique show changes compared to the control textile, where the intensity of peaks, and even some peaks disappear when functionalized, which is possibly due to the appropriate nanoparticle density. Additionally, nanoparticles not only impregnate the surface but also penetrate the core of the textile fiber, allowing for stronger peaks of the nanomaterial to be shown compared to the corresponding groups in the control. Regarding the type of impregnation, changes can also be observed, where samples impregnated by the "Post-Synthesis" method show a lower intensity, which may be associated with a possible loss of nanomaterial on the surface.

The interactions between silver nanoparticles and cotton and polyester textiles can be electrostatic and chemical, favored by the positive charge of silver nanoparticles and the functional groups on the textile surface. In addition, silver nanoparticles can also adsorb onto the surface of the textile due to Van der Waals forces. These interactions can have an impact on the stability of nanoparticles in the textile and their ability to prevent the proliferation of microorganisms.

For the case of cuprous oxide nanoparticles, they have been obtained under alkaline conditions, and therefore the mentioned nanomaterial would have a negative charge due to the presence of cuprate ions on its surface, which in turn favors electrostatic interactions with positively charged groups on the textile surface, such as amino groups in the polyester fiber. In addition, functional groups on the surface of cuprous oxide nanoparticles can chemically interact with chemical groups in the textile. FTIR results of the nanomaterial show the presence of hydroxyl groups on the surface of cuprous oxide nanoparticles, which can interact with carboxyl groups on the surface of the textile, establishing strong chemical bonds.

In zinc oxide, the mechanism is similar, however, the importance of nanomaterial size is worth highlighting, as mentioned in the nanomaterial synthesis section. Better morphology and smaller nanostructures have been obtained with biogenic synthesis methods, so smaller nanoparticles can penetrate more deeply into textile fibers and have a greater surface area, increasing the likelihood of chemical bond formation with functional groups in the textile fibers.

### Assessment of textiles functionalized against the SARS-CoV-2 virus

The method of NP impregnation in the textile fiber plays an important role in the viricidal effects practiced in this research, linked to a better impregnation (in the core and surface of the fiber) which is translated into a higher concentration of nanomaterial. Thus, the "in situ" method shows better SARS-CoV-2 virus elimination results. Firstly, it is possible to compare the cases of textiles impregnated individually with Ag and Cu_2_O NP (Tables 1 and 2), where it is evident that, in both cases, the values of viral load reduction are in higher proportion with the "in situ" impregnation (Ag NP 96.56% and Cu_2_O 99.79%). It should be noted that the cotton content (70%) is an important factor for the impregnation of nanomaterial due to the ease of adhesion since a textile with a higher polyester content does not have a greater porosity opening, becoming a physical barrier that would not allow proper adhesion.

**Table 1:** Viral load reduction using *"*In Situ*"* treatment.

Number of copies removed in situ	Mean	Inoculum	% viral load reduction
Silver (2.411 ppm) *	1.15 ± 0.041	1.20 × 10^7^	95.56
Cuprous oxide (734.132 ppm) *	3.1 ± 0.0055	3.11 × 10^7^	99.79
Fabric control (70% cotton/30% polyester) *	2.97 ± 0.0313	3.11 × 10^7^	95.61

The f-ratio value was 6704.65428.

*The p-value was < .00001. The result was significant at p < .05, using 3 repetitions per group.

**Table 2:** Viral load reduction using the *"Post-Synthesis"* treatment.

Number of copies removed* Post-Synthesis*	Mean	Inoculum	% viral load reduction
Silver (2.411 ppm)*	1.054 ± 0.0508	1.20 × 10^7^	88.00
Cuprous oxide (734.132 ppm)*	6.726 ± 0.0422	7.09 × 10^7^	94.92
Ag/Cu (2.411 ppm/734.132 ppm)*	6.436 ± 0.115	7.09 × 10^7^	90.79
ZnO (398.7 ppm)*	6.886 ± 0.0904	7.09 × 10^7^	97.09
Fabric control (70% cotton/30% polyester)*	2.976 ± 0.0313	3.11 × 10^7^	95.61

The f-ratio value was 6564.40262.

*The p-value was < .00001. The result is significant at p < .05, using 3 repetitions per group.

On the other hand, in an analysis according to the type of nanomaterial (Table 3), it can be evidenced the potential power of SARS-CoV-2 viral load reduction by Cu_2_O NP (99.79%) and ZnO NP (97.09%), values that are above the control (95.61%). However, the textile functionalized with Ag/Cu_2_O hybrid NPs shows a notorious reduction concerning the control (90.79%), most likely linked to an oxidation–reduction process between both types of NPs, which have reacted, leaving without effect potential properties at the level of electrical charges, which play an important role in the reduction of the viral load.

**Table 3:** Viral load reduction using *"*In Situ*"* (Copper) versus *"Post-Synthesis"* (Zinc) treatment.

Number of copies removed IN SITU	Mean	Inoculum	% viral load reduction
Cuprous oxide (734,132 ppm) *	3.1 ± 0.0055	3.11 × 10^7^	99.79
ZnO (398.7 ppm) *	6.886 ± 0.0904	7.09 × 10^7^	97.09

The f-ratio value is 8711.03776. The p-value is < .00001. The result is significant at p < .05, using 3 repetitions per group.

## Discussion

As observed in the in-situ treatment, the viral load reduction values were > 95.56% between the treatments with silver or copper nanoparticles and the fabric control (a fabric used to impregnate the nanoparticles). It was demonstrated that there was a significant difference between the treatments, with the highest value of viral load reduction for the treatment with Cu_2_O NP (99.79%) being statistically significant in comparison with the other treatments (p < 0.05). A previous study using Cu_2_O NP against the Herpes virus showed a value of 83.3% (100 μg/mL) inhibition^59^. Cu_2_O may interfere with viral replication, the creation of ROS (Reactive oxygen species) radicals damaging the viral genome, and the denaturation of viral capsid proteins^59–61^. In the case of the antiviral effect on SARS-CoV-2 on copper-coated surfaces, it has been reported that the virus was not viable after 4 h post-exposure^36^. In addition, a study mentioned that the light-mediated plasmon effect on Cu_2_O NP enhances the antiviral effect^62^, so the use of clothing fabrics or face masks with NPs exposed to sunlight would enhance their antiviral effect. Another study using copper nanofibers in masks against SARS- CoV-2 showed 55% inhibition of viral replication after 48 h of exposure^63^, in contrast to our study with copper treatment that showed a 99.79% viral load decrease effect. Another previous study also showed that copper had good biocompatibility in toxicity and inflammatory response assays, so it is considered a safe metal for use^63^. In the case of Ag NP treatment, the decrease in viral load of SARS-CoV-2 was 95.56%, although with a lower significant effect compared to the control. A study using silver nitrate (AgNO_3_) and silver oxide I (Ag _2_O) showed a potent antiviral effect against influenza A virus and that its effect depended on the solubility of the ion in water, presenting a higher antiviral effect when there is a higher ion solubility^61^. A previous study showed that the use of Ag NP (10 ppm) significantly decreased the number of viral copies (> 10^9^) compared to the control^64^. In contrast, in our study, the Ag nanoparticle treatment decreased viral load by 95.56% in situ and 88% *post-synthesis* at 2.41 ppm.

In Post Synthesis treatment, the highest antiviral effect resulted from using ZnO with a percentage of viral load reduction of 97.09% with a significant difference (p < 0.05) compared to the other treatments. A previous study has shown the antimicrobial effect of ZnO^65^. Another review study indicated that in, In vitro studies, the antiviral effect against coronavirus was through the inhibition of RNA polymerase^66^. Additionally, another study on fabrics impregnated with Zinc showed an inhibitory effect against SARS-CoV-2 and the influenza A virus^67^. Other research using Zn salts demonstrated in In vitro studies an inactivation effect between 63 and 100% of the Herpes virus in a Zn concentration-dependent manner^68^. It has been determined that ZnO can generate free radicals capable of viral damage, inhibit protease and cause damage to viral RNA polymerase^69,70^. A recent study using ZnO NP against SARS-CoV-2 showed a 50% reduction in cytotoxicity caused by the virus in cell culture^71^. Additionally, Zn NP is an ideal antimicrobial and antiviral agent due to its low cytotoxicity^72^.

It was determined that the best treatment for in situ viral load reduction was copper, with 99.79% viral reduction, compared to the best treatment for post-synthesis viral load reduction, which was ZnO, with 97.09%. Therefore, the textile functionalized in situ with CuO NP was the treatment that showed the best effect on the viral load reduction, being statistically significant concerning the control and the other treatments.

## Conclusions

The biogenic synthesis method presents a potential alternative for obtaining nanostructures with defined morphology, high stability, and monodispersity, highlighting the fundamental role of bioactive compounds that act as reducers and stabilizers of precursor metal salts for the formation of silver nanoparticles with diameters of 6 ± 3.7 nm, cuprous oxide nanoparticles of 10 ± 2.92 nm, and zinc oxide nanoparticles of 50 ± 8.3 nm. Furthermore, the obtained nanomaterial was applied in textile impregnation using techniques called 'in situ' and 'post-synthesis', with 'in situ' treatment achieving better nanoparticle impregnation as evidenced by the formation of nanostructures in the core and surface of the textile fiber, while the 'post-synthesis' method shows agglomerations on the surface. On the other hand, it was determined that the type of nanomaterial and textile impregnation are associated with the reduction of viral load (SARS-CoV-2). Textiles impregnated with Cu_2_O nanoparticles showed the best reduction effect on SARS-CoV-2 viral load at 99.79%, followed by textiles functionalized with ZnO nanoparticles (97.09%), which, despite being the 'post-synthesis' method, exhibited good viricidal properties. Therefore, this technology can be employed in various areas of the healthcare sector and for inhibition of bacterial and fungal growth, as previous studies have highlighted the potential effects of these types of nanoparticles.

## Supplementary Information

Supplementary Information.

## Data Availability

The original data are available upon request from the corresponding author: davidasmat88@hotmail.com.
