# Supplementary material for: Biogenic production of silver, zinc oxide, and cuprous oxide nanoparticles, and their impregnation into textiles with antiviral activity against SARS-CoV-2
Source: Sci Rep. 2023 Jun 16;13:9772. doi: 10.1038/s41598-023-36910-x (PMC10275893; doi:10.1038/s41598-023-36910-x)
Supplement: Supplementary file 1 — Supplementary Information. [file 41598_2023_36910_MOESM1_ESM.docx]

**Supporting Information**


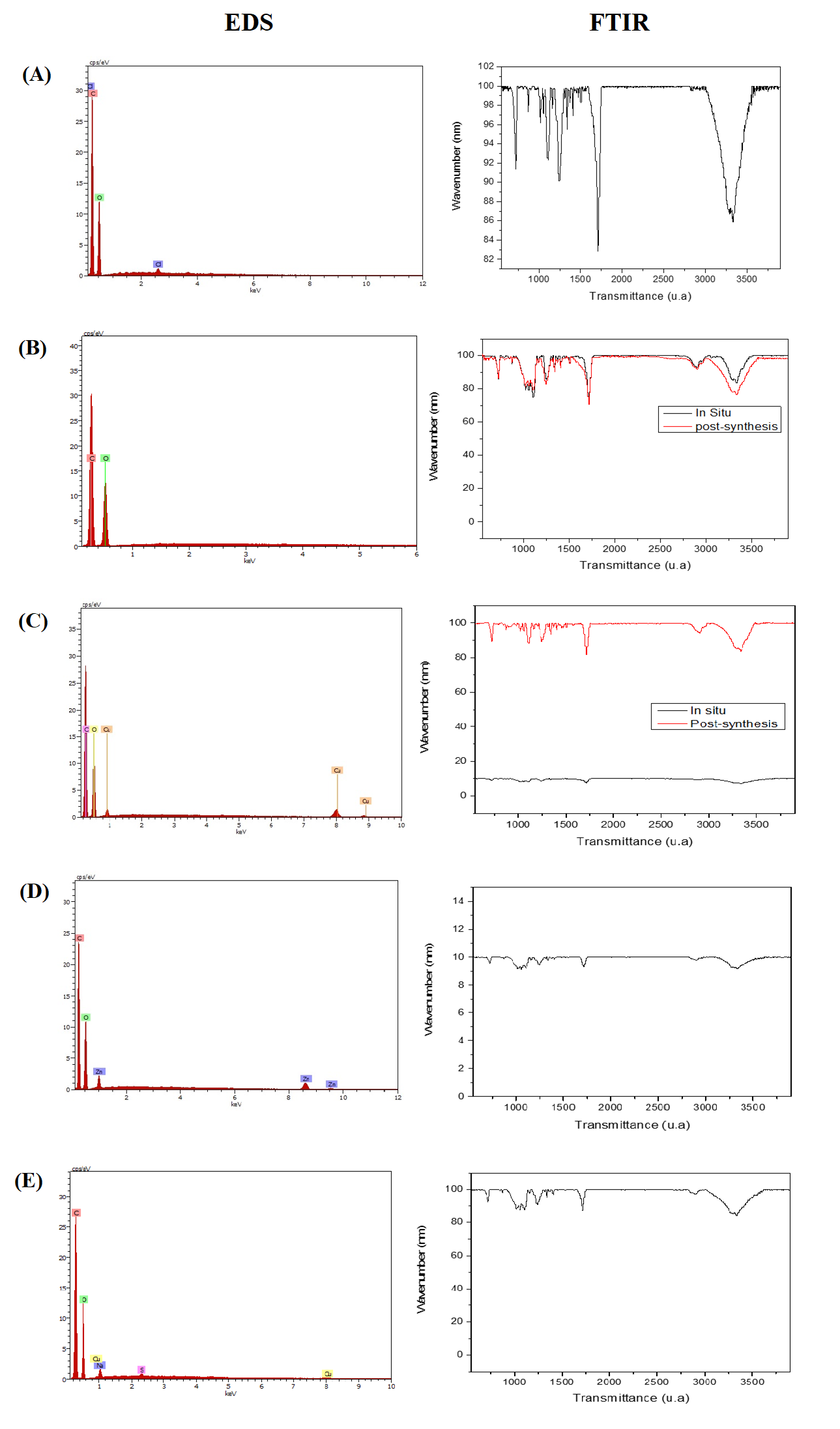


**FIGURE S1.** Characterization by EDS and FTIR of textile samples. (A) Control, (B) Ag NP functionalized, (C) Cu_2_O NP functionalized, (D) ZnO NP functionalized, (E) Ag/Cu_2_O functionalized.
